# Efficacy of “seeking safety” in a Dutch population of traumatized substance-use disorder outpatients: study protocol of a randomized controlled trial

**DOI:** 10.1186/1471-244X-13-162

**Published:** 2013-06-04

**Authors:** Tim Kok, Hein A de Haan, Margreet van der Meer, Lisa M Najavits, Cor AJ DeJong

**Affiliations:** 1 Tactus Addiction Treatment, P.O. box 154, Deventer, 7400 AD, The Netherlands; 2 Nijmegen Institute for Scientist Practitioners in Addiction, P.O. box 6909, Nijmegen, 6503 GK, The Netherlands; 3 Verslavingszorg Noord Nederland, Groningen, P.O. box 1024, 9701 BA, The Netherlands; 4 Boston University School of Medicine, 28 Westbourne Road, Newton Centre, MA, USA; 5 Radboud University Nijmegen, P.O. box 9102, Nijmegen, 6500 HC, The Netherlands

**Keywords:** Seeking safety, Randomized controlled trial, CBT, Substance use, PTSD, Comorbidity

## Abstract

**Background:**

Traumatic experiences and, more specifically, posttraumatic stress disorder (PTSD) are highly prevalent among substance use disorder (SUD) patients. This comorbidity is associated with worse treatment outcomes in substance use treatment programs and more crisis interventions. International guidelines advise an integrated approach to the treatment of trauma related problems and SUD. Seeking Safety is an integrated treatment program that was developed in the United States. The aim of the current study is to test the efficacy of this program in the Netherlands in an outpatient SUD population.

**Methods/Design:**

A randomized controlled trial (RCT) will be used to test the efficacy of Seeking Safety compared to Cognitive Behavioral Therapy (CBT) in a population of SUD outpatients. Each treatment will consist of 12 group sessions. The primary outcome measure will be substance use severity. Secondary outcome measures are PTSD and trauma symptoms, coping skills, functioning, and cognitions. Questionnaires will be administered at the start of treatment, at the end of treatment (three months after the start of treatment) and at follow-up (six months after the start of treatment).

**Discussion:**

This study protocol presents a RCT in which the efficacy of an integrated treatment for comorbid PTSD and SUD, Seeking Safety, is evaluated in a SUD outpatient population compared to CBT. It is expected that the intervention group will show significantly more improvement in substance use severity compared to the control group at end-of-treatment and at follow-up. Furthermore, a lower drop-out rate is expected for the intervention group. If the intervention proves to be effective, it can be implemented. A cost-effectiveness analysis will be conducted to evaluate the two treatments.

**Trial registration:**

The protocol for this study is registered with the Netherlands Trial Register with number NTR3084 and approved by the local medical ethical committee (METC\11270.haa).

## Background

A history of traumatic experiences is very common among substance use disorder (SUD) patients, with estimates ranging from 55%–99% [1,2]. Furthermore, high rates of posttraumatic stress disorder (PTSD) have been found among SUD patients. The rate of drug and/or alcohol dependence in veteran populations varies from 40% to 75% [3,4]. In civilian populations with lifetime PTSD prevalence of SUD’s ranges from 22% to 43%, compared with 2% to 15% in persons without PTSD [5]. Among SUD patients the rate of PTSD varies between 11%–60% for current PTSD and 33%–75% for lifetime PTSD [2,6,7].

These high rates of comorbidity suggest that PTSD and SUD are functionally related. Several pathways have been described in which these two disorders relate to one another, such as the “self-medication hypothesis” in which PTSD precedes SUD [8]. When SUD precedes PTSD, a likely explanation is that substance users often risk hazardous situations to sustain their habit, and therefore experience high levels of physical and psychological trauma. This in turn, increases the chance of developing a PTSD [9]. Finally, there is accumulating evidence that both disorders share neurobiological systems, so that continued substance use may increase an individual’s susceptibility to developing PTSD following a trauma and, vice versa, that PTSD increases the vulnerability for developing SUD [10].

The association between SUD and PTSD is clinically significant, as well as the association with traumatic experiences in general, because trauma exposure and related PTSD symptoms have the potential to affect SUD treatment. These symptoms include re-experiencing the trauma, avoidance, and increased anxiety or hyperarousal. In addition to related symptoms of depression, they interfere with patients’ abilities to adhere to and benefit from substance abuse treatment [11,12]. International guidelines advise to integrate the treatment of SUD and trauma related disorders such as PTSD [13-15].

Seeking Safety is a therapy that was developed in the United States as an integrated treatment for PTSD and SUD [16]. It has also been found a feasible treatment for patients who are subthreshold on these disorders or who have just one or just the other disorder. The treatment is cognitive-behavioral, as well as addressing interpersonal and case management domains. It is present-focused and designed to help patients learn coping skills to attain safety from trauma/PTSD and substance abuse. Seeking Safety has had over 20 published studies (see [17], for a comprehensive review). It has shown to reduce substance use and PTSD symptom severity in comparison with a control group in female patients [18], female adolescents [19], incarcerated women with SUD and PTSD [20] and in veterans in outpatient treatment [21], for example. Various quasi-experimental and non-controlled studies show positive outcomes for Seeking Safety [22-31]. The treatment has shown positive results in the United States and is the only model for PTSD/SUD thus far to impact both PTSD and SUD [17]. The French translation of Seeking Safety has had positive findings and satisfaction in a Canadian study [32]. The Dutch translation, however, has never been studied. Cultural differences in expectancies from health care providers, differences in communicating and general views may cause differences in adherence and efficacy.

### Aims

The aim of this study is to test the efficacy of Seeking Safety versus standard CBT in a Dutch sample of outpatient SUD patients who have experienced at least one traumatic event. As the population under study consists of patients in treatment for substance use, the primary outcome measure is substance use severity. Secondary outcome measures are PTSD and trauma symptoms, coping skills, functioning, and cognitions. We will also assess drop-out and satisfaction.

A second aim of the study is to perform a cost-effectiveness analysis to compare the two treatments.

## Methods

### Study design

For this study a randomized controlled design is used. Semi-structured interviews and self-report questionnaires will be used to measure change over time on variables at three time points: at the start of the treatment (t = 0), at end of the treatment (t = 1) and three months after the end of treatment (follow-up, t = 2). These points in time are chosen to conform to standard evaluation procedures within our facility. Routine outcome monitoring is conducted at completion of treatment for the outpatients or at three months after the start of treatment. The administration of questionnaires for the study will be synchronized with this monitoring. Both Seeking Safety and CBT will consist of weekly group sessions of 1 3/4 hours for 12 weeks (12 sessions total). This design keeps dosage equivalent across the treatment conditions, and has been used successfully in a prior recent RCT of Seeking Safety [21]. Both Seeking Safety and CBT will be offered in closed-group format, as this is the standard in the control condition. Participants will not be financially rewarded for their participation. The results of the study will be presented following the CONSORT guidelines [33].

### Participants

#### Recruitment

All patients that enter group-based weekly therapy are asked to participate during intake. They will receive a letter explaining the purpose of the study, what they can expect and the requirements to participate, and an informed consent. They are asked to read the information carefully and decide on participating within two days.

#### Inclusion criteria

Eligible participants for this study are both male and female patients that enter group-based outpatient substance use treatment. They have to be 18 years of age or older and sufficiently fluent in the Dutch language. In this study we will include all patients who have experienced a traumatic event and have trauma-related symptoms. These are assessed by asking the patients whether or not they have experienced a traumatic event, and have a score of at least 40 on the Self-report Inventory for PTSD (SRIP). Furthermore, patients meet current DSM-IV criteria for substance dependence or abuse and have active substance use in the previous 30 days.

#### Exclusion criteria

Patients will be excluded at intake if they have current uncontrolled psychotic or severe bipolar disorder; or serious danger to self or others (defined as stated intent to commit suicide or an act of violence, with clear intent and plan). These will be determined by a psychologist or a psychiatrist.

#### Randomization

Randomization will be at patient level. The participants will be randomly appointed to either the control group or the experimental group. The care-takers are blind for this randomization. Envelopes will be distributed to the treatment facility and these contain the questionnaires that will be administered at t = 0. After completing the questionnaires patients will be randomized based on a predefined randomization schedule. Randomization will be performed in blocks of 4 and 8 (in random order). The research assistants that administer the questionnaires for t = 1 and t = 2 will be kept blind for this randomization.

### Sample size calculation

The sample size for this study is calculated using a significance level of 95% (p < 0.05), a power of 80% and the same number of patients in the intervention and control group (ratio = 1).

Primary outcome variable is substance use severity as measured with the EuropASI. In recent studies, conducted within the same institution [34] but with a population of inpatients, a mean baseline score of 5.2 was found for the alcohol domain and 4.0 for the drugs domain. We expect the mean baseline scores for the outpatients to be somewhat lower, however not considerably. For comparison of baseline scores on the EuropASI, see [22,23]. For practical reasons we are using the mean of both domains (alcohol and drugs) for the sample size calculation. Thus, the mean score for substance use severity will be somewhat lower than (5.2 + 4.0)/2 = 4.6 with a standard deviation of 2.7. For this sample size calculation we will use a baseline score of 4.0 with the same standard deviation. We expect the substance use severity to improve with 60% [23] to 1.6 (SD = 2.0) at end-of-treatment for the intervention group and 2.8 (SD = 2.0) for the control group. We used PS – Power and Sample Size Calculation to calculate the sample size, which resulted in a sample size of 45 patients in each group. Drop-out rates for this treatment are available for the past years from a management information system and are estimated around 20%. This drop-out rate may seem low compared to the numbers that are known from previous studies in SUD patients. However, we will use this percentage to calculate the number of patients that are needed, but will perform an analysis on drop-out halfway through the study. When the drop-out rates turn out to be higher than expected, an amendment will be proposed to the medical ethical committee to continue including patients for an extended period of time. Because of the calculated drop-out rate, an extra 9 participants will be included in both the intervention and the control group, yielding a total of 108 participants for the randomized controlled trial. Based on treatment entry numbers from previous years, approximately two years will be needed to include all patients. Furthermore, this number of patients seems plausible considering sample sizes in previous studies that show similarity in design [21,22].

### Ethics

The study was approved by the local medical ethical committee (METC\11270.haa).

### Treatment program

#### Intervention

Patients will be randomized to either Seeking Safety or to standard CBT. Seeking Safety was originally designed as an integrated model for patients that have both PTSD or trauma-related symptoms and SUD. The treatment manual has recently been translated into Dutch [35]. Seeking Safety consists of 25 structured topics, which can be conducted in group or individual modality. In this study, it will be conducted in group modality. Topics are evenly divided among cognitive, behavioral and interpersonal coping skills, with a goal of helping patients attain safety in their lives. ‘Safety’ is defined as reduction in substance use and destructive behavior, establishment of a network of supportive people and self-protection from dangers associated with the disorders.

There will be one Seeking Safety topic per session for the duration of three months, making it possible for patients to receive 12 topics. The topics to be used are: Safety; PTSD-Taking Back Your Power; When Substances Control You; Grounding; Healing from Anger; Honesty; Taking Good Care of Yourself; Compassion; Red and Green Flags; Asking for Help; Creating Meaning; and Life Choices.

Patients that attend a minimum of 7 sessions will be considered “minimum dose completers” [36]. Every session is conducted according to a fixed structure, starting with a check-in to find out how each patient is doing and both strengths and problems in coping since the last session. Second, a quotation is discussed followed by a discussion of the hand-outs in which the topic of the session is related to the patients’ life. This is also the place where coping skills are shared and discussed. Each session ends with a check-out where patients are encouraged to share one thing they have learned during the session and to formulate a commitment (homework) for the next session. The sessions will be facilitated by one therapist or social worker. The therapists and social workers all received a two-day training in Seeking Safety. Furthermore, all sessions will be videotaped and a random sample of 20% will be selected for review. Thus, quality and adherence will be monitored and ongoing supervision will be provided.

#### Comparison condition

Cognitive Behavioral Therapy (CBT) is an evidence-based cognitive behavioral, manual-based model focused on reducing substance use. It includes relapse prevention and motivational interviewing in which positive reinforcement and enhancing self-efficacy among others, are used to attain behavioral change in the patient [37]. CBT is offered once a week for a period of three months in a closed group.

### Data collection

Patients in both treatment conditions will be assessed identically: at the start of treatment (baseline), at the end of treatment (after three months) and at follow up (six months after start of treatment). Patients will be assessed by research assistants at these time points. Under their supervision patients will fill out most of the questionnaires in an online environment in which the data is linked to their electronic patient file.

### Instruments and outcome

The primary outcome in this study is substance use severity at end of treatment as measured with the EuropASI. This measure will provide a composite score for alcohol and drugs which incorporates the number of days of use in the previous 30 days, amount of money spent on drugs or alcohol, perceived problems and burden of problems associated with substance use. The composite score for alcohol use severity will be used as the primary outcome measure for patients with alcohol use disorder and the composite score for drug use severity will be used for patients with substance use disorder according to the DSM-IV. Secondary outcome measures are the other measures listed below.

### European addition severity index (EuropASI)

The EuropASI is the European adaptation of the fifth edition of the addiction severity index [38]. It is a semi-structured interview that gives a multidimensional profile of the substance-dependent individual and the severity of the addiction. It covers seven main problem areas (medical, employment, alcohol, drug, legal, psychiatric, and family and social) that are most commonly affected by substance abuse. Scoring is based on two indices for each problem area: firstly, the interviewer’s severity rating, in which the interviewer indicates the severity of the patient’s problems and his or her present need for additional treatment on a 9-point scale. Secondly, a composite score (range 0 - 1), an arithmetically based indicator of current (30 day) problem severity, is obtained. Composite scores for the EuropASI are computed with a strategy similar to the one used in the ASI [39].

### Obsessive compulsive drinking scale (OCDS)

The OCDS is a quick and reliable self-rating instrument that provides a score that measures cognitive aspects of craving of alcohol or drugs during the past seven days [40]. The short version of the scale [41] that is used in the current study consists of 5 items which can be scored on a 0-4 scale, where a higher score indicates higher obsessions and compulsions regarding drugs or alcohol.

### Self report inventory PTSD (SRIP)

The SRIP is a self-report measure of PTSD [42]. The duration of this measure is five minutes, which makes it very time-efficient. The SRIP contains 22 items, based on the DSM-IV-TR criteria for PTSD. The items are scored on a 4-point Likert scale, varying from 1 ‘not at all’ to 4 ‘often’, and indicates the intensity of the PTSD-symptoms in the past month. A total-score of 52 or higher indicates a PTSD-diagnosis. The SRIP uses the three subscales described in de DSM-IV-TR criteria: ‘Intrusion’, ‘Avoidance’, and ‘Hyper arousal’. Besides this, the 22 items are subdivided in four factors: ‘Emotional numbing’, ‘Avoidance’, ‘Intrusion’, and ‘Sleeping problems’. The test does not contain trauma-specific items, therefore the SRIP is suitable for any population. Research provides evidence for a high reliability of this measure. Hovens [43] found, with the Clinician Administered PTSD Scale (CAPS) [44] as criterion, an internal consistence reliability of the total score between .90 and .94.

### Trauma symptom checklist (TSC-33)

The TSC-33 is a research measure that evaluates symptomatology in adults associated with childhood or adult traumatic experiences. It measures aspects of posttraumatic stress and other symptom clusters found in some traumatized individuals. It does not measure all 17 criteria of PTSD, and should not be used as a complete measure of that construct [45]. It is included in the current study to measure the aspects of psychological effects of traumatic experiences in addition to the standard DSM-IV criteria of PTSD.

### Utrecht coping list (UCL)

The UCL [46] is a self-report instrument to measure the way people deal with problems or stressful situations. The questionnaire consists of 47 items divided into 7 subscales measuring seven different coping strategies: 1. ‘Active problem solving’, 2. ‘Palliative responses’, 3. ‘Avoidance and passive expectancy’, 4. ‘Seeking social support’, 5. ‘Depressive reaction pattern’, 6. ‘Expressing emotions’ and 7. ‘Comforting cognitions’. Each item can be answered with ‘seldom or never’, ‘sometimes’, ‘often’ and ‘very often’. A total score is obtained by adding the scores of all items together.

It is important to note, that although Seeking Safety emphasizes coping skills, we do not expect the scores of all patients to improve on every subscale of the UCL. Some of the ideas behind Seeking Safety are actually aimed at helping patients to avoid certain situations or feelings. This is not considered to be good or bad. The purpose of this study is to explore changes in coping styles and relate these changes to Seeking Safety.

### EuroQol-5D (EQ-5D)

For the overall quantification of health status as a single index we will use the standard EQ-5D classification system developed by the EuroQol Group [47]. The EQ-5D is one of the three widely used multi-attribute systems available to determine health state preferences (utilities). The EQ-5D classification describes health status according to five attributes (five questions): mobility, self-care, usual activities, pain/discomfort and anxiety/depression. Each attribute has three levels, i.e., ‘no problems’ (1), ‘some problems’ (2) and ‘severe problems’ (3). Health state descriptions are constructed by taking one level for each attribute (e.g., 11111 represents the highest health status). Theoretically this set of attributes and the levels of the EuroQol-5D instrument allow for 243 (35) different health-status descriptions. Based on the descriptive classification of the EQ-5D system a preference index (utility) can be estimated that expresses the overall preference of the classified health status. These utility figures are required to calculate Quality-Adjusted Life Years (QALYs) for the cost-utility analysis [48].

### Sheehan disability scale (SDS)

The SDS [49] is a brief self-report measure that was developed to assess functional impairment in three inter-related domains: work/school, social and familiy life. The patient rates the extent to which problems in these areas are present on a 10 point visual analogue scale and can thus be used to measure functioning.

### Statistical analyses

All analyses will be executed using SPPS for Windows version 18.0. Frequency tables will be provided for all baseline, end of treatment and follow-up variables. Descriptive statistics will include mean, median, interquartile range, numbers and percentages of patients, as appropriate. If applicable, 95% confidence intervals will be given. To compare differences between groups (Seeking Safety vs CBT) *t*-test or mann whitney U tests will be used in case of continuous variables, as appropriate. For categorical variables chi-squared tests will be used. Repeated measures analyses (mixed models in SPSS) will be used to test the differences between the treatment conditions over time in severity of substance use problems as measured by the EuropASI severity score (pre, post and follow-up). The data will be tested for normality and transformed as appropriate when the data are skewed. Analyses will be based on the intent-to-treat principle. Participants have to attend at least one session to be considered part of this group. Differences regarding gender will be analysed and when appropriate, gender will be included in the analyses as a confounder or effect modifier. Multiple Imputation will be used for missing data. Effect sizes will be calculated to facilitate comparison of improvement between the two groups. Linear regression analyses will be used to determine the predictors of change in substance use and trauma symptoms. Also, amount of prior psychiatric treatment will be used in the analysis as a covariate.

### Cost-effectiveness analysis

To make a comparison between the two treatments, the cost of these treatments (total costs of staff and materials) will be related to the benefits of the treatment by performing a cost-effectiveness analysis. A cost-effectiveness analysis requires a numerical estimate of the magnitude of the effects of an intervention on health outcomes. The primary outcome measure of this study will be used for this purpose. Cost will be divided by effects to calculate the cost-effectiveness ratio (C/E). The statistics of interest in the economic evaluation of health care interventions is the incremental cost-effectiveness ratio, which is defined as the difference in cost between Seeking Safety and CBT. To quantify uncertainty in the estimated incremental cost-effectiveness ratio, confidence intervals will be calculated. In addition, a special variant of economic analysis is planned, i.e., cost-utility analysis, to incorporate the preferences (utilities) of the participants considering their own health status at the start of treatment and after follow-up. Based on these preferences combined with life expectancy figures, calculation of so-called QALYs is applicable. A general measure to express the benefits of the two different treatment strategies will be calculated by subtracting the number of quality-adjusted life years computed after follow-up from the number of QALYs computed at baseline. Subsequently, quality-adjusted life years can be combined with the cost of each treatment strategy to arrive at the cost per QALY gained [48,50].

## Discussion

This study protocol presents the design of an RCT evaluating the efficacy of the Seeking Safety treatment program in the Netherlands. The goal of the treatment is to reduce substance use and PTSD-related symptoms. Recently Najavits and Hien have summarized the literature on Seeking Safety, which shows consistent improvements when Seeking Safety is used, even among highly complex and severe patients [17]. Therefore it can be expected that an integrated treatment approach for substance use and trauma-related problems can be very effective. Although Seeking Safety has already shown positive results and is considered to be the first choice of evidence-based treatment for comorbid SUD and PTSD, there are several reasons why this RCT should be conducted.

Firstly, the studies from which the results have been used as evidence for the efficacy of Seeking Safety, are largely conducted in the United States. SUD populations in the United States and Europe or the Netherlands are very similar as general characteristics are concerned. However, communication styles differ between countries, because of cultural differences. Also, the Netherlands are known for its liberal policies and views towards several issues, for example drugs. These differences could have influence on the way patients perceive and adhere to Seeking Safety. The treatment uses a book with handouts for patients and guidance for therapists. This book and its handouts have been translated into Dutch in 2010 [35], however, it is important to test the efficacy of the Dutch version for reasons mentioned above. A natural choice for a comparision condition is CBT, which is considered the gold-standard treatment for SUD alone.

Secondly, the majority of studies focused on female populations of SUD patients. While this group in particular may benefit from Seeking Safety, studies on male subpopulations have emerged over the last few years [51]. In general, there are approximately twice as many male patients than female patients in substance use treatment [52] and it is therefore interesting to see what the effect of Seeking Safety is on a population where men are the majority and Seeking Safety groups are mixed-gender. The effect of gender in response to PTSD-focused treatment has not yet been systematically studied [13]. Therefore, in the current study gender will be studied as a possible confounder or modifier of the effect of Seeking Safety.

Thirdly, while previous studies on Seeking Safety have mostly been focused on patients with PTSD, in this study patients will also be included if they have experienced a traumatic event and present with some PTSD symptoms, but not necessary with full-blown PTSD. That way, the possible benefits of this treatment will be available for a substantial larger group of patients. A possible limitation of this procedure is that self-reported PTSD-related symptoms will be used to include patients in the study. Because the sample will consist of SUD patients, a proportion of the symptoms they report, may be due to substance use or withdrawal (e.g. sleep problems, difficulties concentrating). The symptoms of hyperarousal could be independent of any traumatic experience. For the purpose of this study, however, this is not a problem. There may be instances where a patient is included in the study while his symptoms are primarily related to substance use, but we still expect this patients to benefit from Seeking Safety in alleviating his symptoms.

The aim of this RCT is to test the efficacy of Seeking Safety versus CBT in an outpatient population of SUD patients. The design of the study is based on the fact that both Seeking Safety and CBT are evidence-based models, but CBT does not address trauma or PTSD. We expect that a majority of the patients may benefit from a treatment that focuses on both trauma and substance use. If the intervention group is more successful in reducing substance use and PTSD symptoms, it can be argued that Seeking Safety should be offered for patients presenting with trauma issues.

If either treatment proves to be more effective, cost-effectiveness must also be taken into account because scarcity of resources is a permanent feature of mental health care and substance use treatment in particular. It is well documented that patients that experienced trauma are in general higher users of services in the health care system [53]. However, it is difficult to determine for each hospitalization or crisis whether or not underlying PTSD is its direct cause, which makes economic evaluations difficult. Domino et al. [54] attempted to shed light on this subject by analyzing total service costs of women with mental health and substance abuse disorders who have experienced trauma. One group of women was enrolled in an intervention condition, which provided comprehensive, integrated and trauma-informed services. The other women received care-as-usual. The results showed that the intervention modestly improved clinical outcomes, especially drug use and trauma outcomes, and that there were no differences in cost between the intervention and the comparison condition. This provides support for the notion that integrated and trauma-focused interventions can be effective treatments for SUD patients with traumatic experiences, with no significant extra cost.

### Implications for practice

If Seeking Safety turns out to be an effective treatment for the population under study, it could be implemented broadly within the outpatient setting of addiction treatment facilities. Cost-effectiveness analyses will be used to inform policy makers about the possible advantages of implementing Seeking Safety versus CBT.

## Competing interest

No external funding has been awarded for this study. All authors declare they have no conflict of interest, except Lisa Najavits, who does report a conflict of interest as she is the author and developer of Seeking Safety.

## Authors’ contributions

TK was primarily responsible for the design of the study and drafted the manuscript. HdH participated in the design of the study and helped to write the manuscript. MvdM wrote the initial idea for the study protocol and helped improve it during the process. LN participated in the design of the study, and was involved in editing and scientific feedback on the manuscript. Finally, CdJ participated in the design of the study, supervised the process and made contributions to the content of the manuscript. All authors read and approved the final manuscript.

## Pre-publication history

The pre-publication history for this paper can be accessed here:

http://www.biomedcentral.com/1471-244X/13/162/prepub
